# Analysis of DTI-Derived Tensor Metrics in Differential Diagnosis between Low-grade and High-grade Gliomas

**DOI:** 10.3389/fnagi.2017.00271

**Published:** 2017-08-08

**Authors:** Liang Jiang, Chao-Yong Xiao, Quan Xu, Jun Sun, Huiyou Chen, Yu-Chen Chen, Xindao Yin

**Affiliations:** ^1^Department of Radiology, Nanjing First Hospital, Nanjing Medical University Nanjing, China; ^2^Department of Radiology, Brain Hospital Affiliated to Nanjing Medical University Nanjing, China

**Keywords:** diffusion tensor imaging, magnetic resonance imaging, low-grade gliomas, high-grade gliomas, gliomas grading

## Abstract

**Purpose:** It is critical and difficult to accurately discriminate between high- and low-grade gliomas preoperatively. This study aimed to ascertain the role of several scalar measures in distinguishing high-grade from low-grade gliomas, especially the axial diffusivity (AD), radial diffusivity (RD), planar tensor (Cp), spherical tensor (Cs), and linear tensor (Cl) derived from diffusion tensor imaging (DTI).

**Materials and Methods:** Fifty-three patients with pathologically confirmed brain gliomas (21 low-grade and 32 high-grade) were included. Contrast-enhanced T1-weighted images and DTI were performed in all patients. The AD, RD, Cp, Cs, and Cl values in the tumor zone, peritumoral edema zone, white matter (WM) adjacent to edema and contralateral normal-appearing white matter (NAWM) were calculated. The DTI parameters and tumor grades were statistically analyzed, and receiver operating characteristic (ROC) curve analysis was also performed.

**Results:** The DTI metrics in the affected hemisphere showed significant differences from those in the NAWM, except for the AD values in the tumor zone and the RD values in WM adjacent to edema in the low-grade groups, as well as the Cp values in WM adjacent to edema in the high-grade groups. AD in the tumor zone as well as Cs and Cl in WM adjacent to edema revealed significant differences between the low- and high-grade gliomas. The areas under the curve (Az) of all three metrics were greater than 0.5 in distinguishing low-grade from high-grade gliomas by ROC curve analysis, and the best DTI metric was Cs in WM adjacent to edema (Az: 0.692).

**Conclusion:** AD in the tumor zone as well as Cs and Cl in WM adjacent to edema will provide additional information to better classify gliomas and can be used as non-invasive reliable biomarkers in glioma grading.

## Introduction

Gliomas are the most common primary brain tumors with an annual incidence of 5/100,000 individuals (Ricard et al., 2012; Louis et al., 2016; Miranda-Filho et al., 2017). High-grade gliomas [World Health Organization (WHO) grade III tumors and grade IV tumors] account for 75% of primary brain tumors in adults (Louis et al., 2016). Glioma grading is important for the determination of appropriate treatment methods and prognostic evaluations (Sugahara et al., 1999; Law et al., 2003; Inoue et al., 2005; Kang et al., 2011; Ricard et al., 2012). Different glioma grades require different treatments, including maximal surgical resection, radiotherapy and adjuvant chemotherapy. Moreover, the prognosis of different grade gliomas also varies. Patients suffering from glioblastoma multiforme (GBM, grade IV) have a median survival of only 15 months while those with anaplastic astrocytoma have a median survival of ≤3 years (Chen J. et al., 2012; Westermark, 2012; Omuro and DeAngelis, 2013; Thakkar et al., 2014). The 5-year survival of patients with GBM is approximately 10% (Omuro and DeAngelis, 2013). Grade III tumors have a better prognosis than GBM but may progress and follow a similar course (Omuro and DeAngelis, 2013). Therefore, it is important to accurately distinguish the glioma grade before an operation.

Magnetic resonance imaging (MRI) plays a pivotal role in brain glioma diagnosis. Conventional MRI sequences with T2, FLAIR and contrast-enhanced T1-weighted sequences can illuminate the size, shape, lesion structure, and enhancement patterns. Additionally, while different grade gliomas may have similar enhancement patterns, both low- and high-grade gliomas can show extensive edema on MRI (Stadlbauer et al., 2006). Differential glioma grade diagnosis is still challenging. Diffusion tensor imaging (DTI) can provide extensive information regarding the preferential diffusion of water in brain tissue, which reveals quantitative measures of the water’s molecular motion in a three-dimensional space (Basser and Pierpaoli, 2011). Three eigenvectors and three eigenvalues are used to represent the main diffusion directions and magnitudes, respectively, by combining the eigenvalues, λ_1_, λ_2_, and λ_3_ (Basser et al., 1994). Multiple tensor metrics, including average diffusion coefficient (ADC), fractional anisotropy (FA), mean diffusivity (MD), axial diffusivity (AD), radial diffusivity (RD), planar tensor (Cp), spherical tensor (Cs), and linear tensor (Cl) can be calculated from DTI (Basser and Pierpaoli, 2011). However, although previous studies mainly demonstrated the role of ADC, FA, and MD in glioma imaging (Stadlbauer et al., 2006; Inano et al., 2014; Davanian et al., 2017), few have investigated the value of the rest of the scalar parameters, including the AD, RD, Cp, Cs, and Cl values in gliomas grade diagnosis. ADC, FA, and MD in the tumor zone are useful for distinguishing glioma grades. However, for the peritumoral edema zone and infiltrated areas, other DTI metrics (AD, RD, Cp, Cs, and Cl) seem promising in analyzing glioma subregions, and might even be superior to the “classical” FA and MD values (Wang S. et al., 2009; Wang W. et al., 2009; Wang et al., 2011; Cortez-Conradis et al., 2013).

The current study aimed to investigate a comprehensive evaluation of the diagnostic value of the tensor metrics between high- and low-grade gliomas, especially the AD, RD, Cp, Cs, and Cl values. Differentiations between the five metrics were compared in affected hemispheres [tumor zone, peritumoral edema zone, and white matter (WM) adjacent to edema] and normal-appearing white matter (NAWM) between the low- and high-grade groups. Moreover, the sensitivity of these DTI metrics in glioma grading was also evaluated.

## Materials and Methods

### Patients

A total of 53 inpatients (33 males, 20 females; age range: 20–70 years; mean age: 49.6 years) with pathological glioma confirmation were enrolled between May 2014 and August 2016 at the Brain Hospital Affiliated with Nanjing Medical University. Exclusion criteria included corticosteroid or antibiotic treatment, lesions with calcification and/or hemorrhage, previous brain surgery and gliomas with no edema. A total of 21 patients had a histologically verified low-grade glioma (1 grade I and 20 grade II), and 32 patients had a histologically verified high-grade glioma (16 grade III and 16 grade IV), according to the World Health Organization (WHO) classification (Louis et al., 2016). Each patient received contrast-enhanced (CE) T1-weighted and DTI preoperatively.

All the subjects provided written informed consent before their participation in the study protocol, which was approved by The Research Ethics Committee of the Nanjing Medical University.

### Magnetic Resonance Imaging

All studies were performed on the 3.0 T MR system (Magnetom Verio, Siemens Medical Solutions, Erlangen, Germany). A T1 inversion recovery (T1-IR) sequence was performed axially with the following parameters: repetition time (TR)/echo time (TE) = 250/2.67 ms, matrix size = 307 × 384, field of view (FOV) = 220 mm × 220 mm, slice thickness = 6 mm, and slice gap = 1.5 mm. A T2-weighted sequence was performed axially with the following parameters: TR/TE = 3000/102 ms, matrix size = 307 × 384, FOV = 220 mm × 220 mm, slice thickness = 6 mm, and slice gap = 1.5 mm. After gadopentetate dimeglumine contrast medium injection, a three-dimensional isotropic spoiled gradient echo (SPGR) sequence was acquired with the following parameters: TR/TE = 3000/102 ms, matrix size = 307 × 384, FOV = 220 mm × 220 mm, and slice thickness = 6 mm.

Diffusion tensor imaging was performed axially using a single-shot echo-planar imaging (EPI) sequence with the following parameters: TR/TE = 9000/104 ms, matrix size = 128 × 128, FOV = 230 mm × 230 mm, slice thickness = 2.5 mm, slice gap = 0 mm, diffusion gradient encoding in 30 directions, and *b* = 1000 s/mm^2^. The acquisition time was 5 min and 8 s. In all cases, DTI was performed before the gadolinium injection.

### MRI Data Analysis

We transferred the diffusion tensor data to an independent workstation for post-processing using a native station. Three principal eigenvalues (λ_1_, λ_2_, λ_3_) were calculated for each voxel. Maps for AD, RD, Cp, Cs, and Cl were available. The AD, RD, Cp, Cs, and Cl values were calculated as follows:

$$\begin{array}{ccc} AD=\lambda_{1} & RD=\frac{\lambda_{2}+\lambda_{3}}{2} & Cp=\frac{2\left(\lambda_{2}-\lambda_{3}\right)}{\lambda_{1}+\lambda_{2}+\lambda_{3}}\\ Cs=\frac{3\lambda_{3}}{\lambda_{1}+\lambda_{2}+\lambda_{3}} & & CI=\frac{\left(\lambda_{1}-\lambda_{2}\right)}{\lambda_{1}+\lambda_{2}+\lambda_{3}} \end{array}$$

Regions of interest (ROIs) were generated under the guidance of a neuroradiologist (Chao-Yong Xiao and Xindao Yin) with more than 15 years of clinical experience.

For each case, three ROIs with a maximum average size of 10 mm^2^ were drawn at four zones: the tumor zone (tumor strong signal enhanced area on the CE T1-weighted images), peritumoral edema zone (edema most adjacent to the tumor zone), WM adjacent to edema (white matter zone most adjacent to the distant edema) and contralateral hemisphere NAWM. These ROIs were put onto the abovementioned DTI maps automatically. Average AD, RD, Cp, Cs, and Cl values were calculated in ROIs.

### Statistical Analysis

The data were analyzed using SPSS software (version 19.0; Chicago, IL, United States). For comparisons between the results, two-sided paired and *t*-tests were used. The data are presented as the mean value ± SD, and significance was indicated by *P*-values < 0.05. Paired *t*-test was used to compare the AD, RD, Cp, Cs, and Cl values in the affected hemisphere (tumor zone, peritumoral edema zone, and WM adjacent to edema) with those in NAWM from low- and high-grade groups. Independent sample *t*-test was performed to compare the AD, RD, Cp, Cs, and Cl values of the tumor zone, peritumoral edema zone, WM adjacent to edema, and NAWM between low- and high-grade gliomas. The metrics of *P* < 0.05 were divided into five groups by ascending order. The cases of the groups were estimated by using ROCKIT software. ROC curves were drawn with the Excel 2010 software.

## Results

**FIGURE 1:**
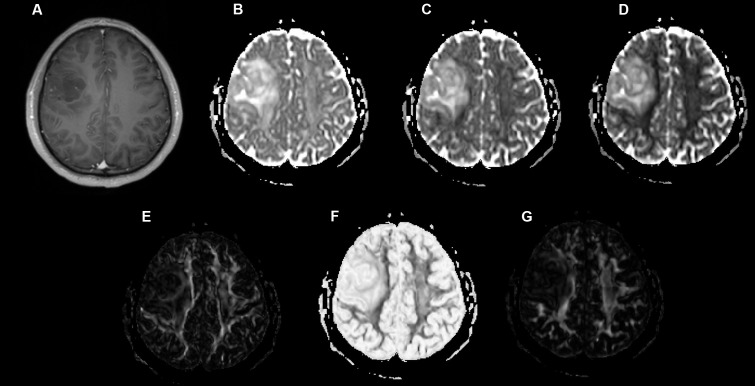
A 49-year-old-man with a histologically verified oligodendroglioma (grade II). **(A)** There was no obvious enhancement within the right frontal lobe mass on the axial contrast-enhanced T1-weighted image. **(B–G)** DTI that included λ1 **(B)**, λ2 **(C)**, λ3 **(D)**, Cp **(E)**, Cs **(F)**, and Cl **(G)** maps demonstrating the lesion.

**FIGURE 2:**
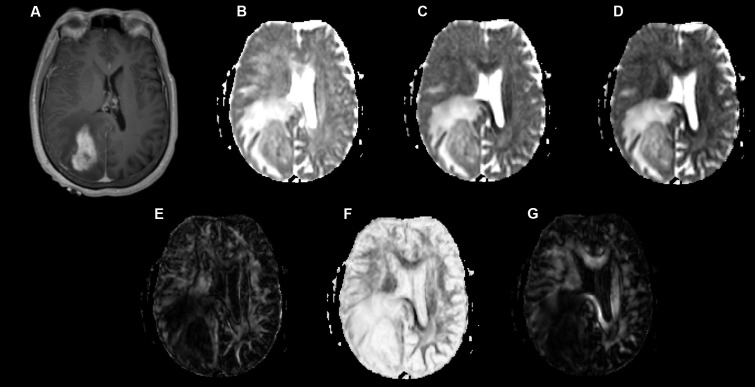
A 58-year-old-woman with a histologically verified glioblastoma multiforme (grade IV). **(A)** An axial T1-weighted image after contrast administration shows an enhanced mass with the right parietal-occipital lobe. **(B–G)** DTI that included λ1 **(B)**, λ2 **(C)**, λ3 **(D)**, Cp **(E)**, Cs **(F)** and Cl **(G)** maps demonstrating the lesion.

Examples of a WHO grade II astrocytoma and a grade IV GBM with their respective λ_1_, λ_2_, λ_3_, Cp, Cs, and Cl maps are shown in **Figures 1**, **2**.

**Table 1** shows different DTI metrics within various zones of the low- and high-grade groups. The AD value differences in the tumor zones between the low- and high-grade groups were statistically significant (*t* = 2.048; *p* = 0.046). Significant Cs and Cl value differences in the WM adjacent to edema were also observed between the two groups (*t* = -2.171, *p* = 0.035; and *t* = 2.324, *p* = 0.024, respectively). No significant differences were seen in the other metrics.

**Table 1:** Comparison of different DTI metrics within various zones between low-grade and high-grade groups.

		Tumor zone	Peritumoral edema zone	WM adjacent to edema	NAWM
AD	Low-grade	1.35 ± 0.36	1.70 ± 0.32	1.18 ± 0.18	1.31 ± 0.15
	High-grade	1.16 ± 0.34^∗^	1.77 ± 0.32	1.10 ± 0.17	1.30 ± 0.13
RD	Low-grade	1.09 ± 0.30	1.35 ± 0.30	0.58 ± 0.11	0.50 ± 0.16
	High-grade	0.93 ± 0.35	1.41 ± 0.32	0.65 ± 0.17	0.49 ± 0.09
Cp	Low-grade	0.08 ± 0.04	0.07 ± 0.04	0.19 ± 0.06	0.21 ± 0.08
	High-grade	0.11 ± 0.07	0.10 ± 0.06	0.19 ± 0.06	0.20 ± 0.07
Cs	Low-grade	0.86 ± 0.07	0.87 ± 0.07	0.60 ± 0.14	0.48 ± 0.10
	High-grade	0.82 ± 0.10	0.82 ± 0.14	0.68 ± 0.12^∗^	0.50 ± 0.11
Cl	Low-grade	0.06 ± 0.04	0.07 ± 0.04	0.22 ± 0.12	0.31 ± 0.10
	High-grade	0.07 ± 0.07	0.06 ± 0.03	0.15 ± 0.08^∗^	0.31 ± 0.10


*Data are presented as mean ± SD. ^∗^*p* < 0.05. AD, axial diffusivity; RD, radial diffusivity; Cp, planar tensor; Cs, spherical tensor; Cl, linear tensor; WM, white matter; NAWM, normal-appearing white matter.*

**Tables 2**, **3** present comparisons of the results of each tensor metric in the low- and high-grade groups, respectively. In the low-grade groups, there was a statistically significant difference for the DTI metrics in the affected hemispheres compared with the NAWM, except for the AD values in the tumor zones and the RD values in the WM adjacent to edema. In the high-grade groups, *post hoc* tests also showed significant differences in almost all tensor metrics, except for the Cp values in the WM adjacent to edema.

**Table 2:** Comparison of different DTI metrics among various zones in low-grade groups.

	Tumor zone VS NAWM		Peritumoral edema zone VS NAWM		WM adjacent to edema VS NAWM	
			
	*T*-value	*P*-value	*T*-value	*P*-value	*T*-value	*P*-value
AD	0.573	0.573	4.817	0.000^∗^	-2.442	0.024^∗^
RD	9.546	0.000^∗^	10.010	0.000^∗^	2.032	0.056
Cp	-6.780	0.000^∗^	-7.407	0.000^∗^	-1.102	0.028^∗^
Cs	14.173	0.000^∗^	12.621	0.000^∗^	3.676	0.001^∗^
Cl	-11.583	0.000^∗^	-9.769	0.000^∗^	-2.652	0.015^∗^


*^∗^*p* < 0.05. AD, axial diffusivity; RD, radial diffusivity; Cp, planar tensor; Cs, spherical tensor; Cl, linear tensor; WM, white matter; NAWM, normal-appearing white matter.*

**Table 3:** Comparison of different DTI metrics among various zones in high-grade groups.

	Tumor zone VS NAWM		Peritumoral edema zone VS NAWM		WM adjacent to edema VS NAWM	
			
	*T*-value	*P*-value	*T*-value	*P*-value	*T*-value	*P*-value
AD	-2.193	0.036^∗^	8.239	0.000^∗^	-4.978	0.000^∗^
RD	6.889	0.000^∗^	15.895	0.000^∗^	5.212	0.000^∗^
Cp	-5.846	0.000^∗^	-6.609	0.000^∗^	-1.139	0.264
Cs	12.091	0.000^∗^	8.220	0.000^∗^	7.988	0.000^∗^
Cl	-12.892	0.000^∗^	-15.168	0.000^∗^	-7.142	0.000^∗^


*^∗^*p* < 0.05. AD, axial diffusivity; RD, radial diffusivity; Cp, planar tensor; Cs, spherical tensor; Cl, linear tensor; WM, white matter; NAWM, normal-appearing white matter.*

**FIGURE 3:**
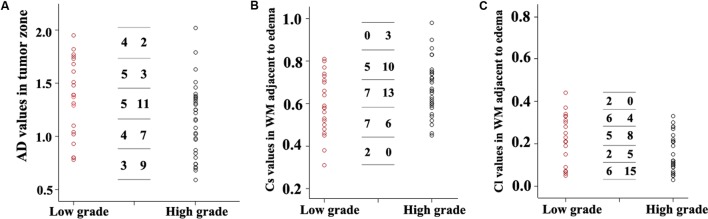
Scatter plots of the AD values **(A)** in the tumor zone and Cs **(B)** and Cl values **(C)** in WM adjacent to edema for all individual low- and high-grade glioma cases. The low-grade cases are shown as red circles and the high-grade are shown as black circles. A transverse line representing the values was divided into five groups. For each DTI metric, the first column number represents the subgroups of low-grade grade glioma cases, while the second column represents the subgroups high-grade glioma subgroups cases.

**FIGURE 4:**
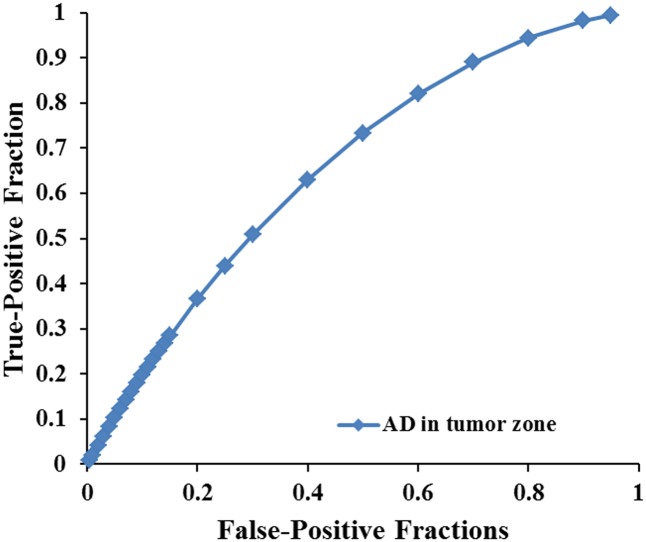
The area under the ROC curve value for AD values in the tumor zone was 0.659 in distinguishing low-grade from high-grade gliomas.

**FIGURE 5:**
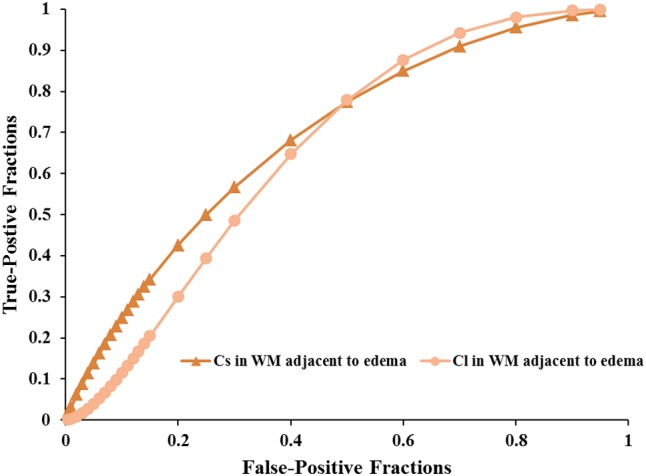
The area under the ROC curve values for Cs and Cl values in WM adjacent to edema were 0.692 and 0.663 in distinguishing low-grade from high-grade gliomas, respectively.

Scatterplots of the AD values in the tumor zone as well as the Cs and Cl values in the WM adjacent to edema were plotted for the low- and high-grade groups in **Figure 3**, which showed the basis of the original ROC curve data (**Table 4**). **Table 5** shows the ROC curve analysis results for the AD values in the tumor zone as well as the Cs and Cl values in the WM adjacent to edema. In the ROC curve analysis, the Az of the all three metrics were greater than 0.5, and Cs in WM adjacent to edema was best suited in distinguishing low- from high-grade gliomas (Az: 0.692) (**Figures 4**, **5**).

**Table 4:** Original data of ROC curve of three DTI metrics.

False-Positive Fractions Of three DTI metrics	True-positive Fractions		
	
	AD in tumor zone	Cs in WM adjacent to edema	Cl in WM adjacent to edema
0.005	0.0097	0.0167	0.0007
0.01	0.0201	0.0323	0.0025
0.02	0.0411	0.0613	0.0085
0.03	0.0619	0.0885	0.0169
0.04	0.0823	0.1142	0.0273
0.05	0.1024	0.1387	0.0393
0.06	0.1222	0.1622	0.0525
0.07	0.1415	0.1848	0.0668
0.08	0.1606	0.2067	0.0821
0.09	0.1793	0.2279	0.0981
0.10	0.1976	0.2484	0.1148
0.11	0.2157	0.2683	0.1321
0.12	0.2335	0.2876	0.1498
0.13	0.2509	0.3064	0.1679
0.14	0.2681	0.3248	0.1863
0.15	0.2850	0.3426	0.2049
0.20	0.3654	0.4257	0.3000
0.25	0.4397	0.4999	0.3946
0.30	0.5083	0.5666	0.4854
0.40	0.6302	0.6812	0.6480
0.50	0.7334	0.7745	0.779
0.60	0.8196	0.8501	0.8765
0.70	0.8898	0.9099	0.9423
0.80	0.9441	0.9550	0.9804
0.90	0.9820	0.9856	0.9969
0.95	0.9940	0.9953	0.9995


**Table 5:** Results of the ROC curve analysis in distinguishing low-grade from high-grade gliomas.

Different zones	DTI metrics	*a*	*b*	Az	*SE* (Az)	*P*-value	95% CI (Az)	
Tumor zone	AD	0.623	1.150	0.659	0.080	0.005	0.494	0.798
WM adjacent to edema	Cs	0.754	1.118	0.692	0.077	0.000	0.529	0.825
	Cl	0.769	1.537	0.663	0.088	0.009	0.478	0.814


*a, intercept; b, slope; Az, area under the ROC curve; SE, standard error; CI, confidence interval; WM, white matter.*

## Discussion

This study mainly demonstrated that most DTI metrics in the affected hemisphere were significantly different from those in the NAWM, except for the AD values in the tumor zone and the RD values in WM adjacent to edema in the low-grade groups along with the Cp values in WM adjacent to edema in the high-grade groups. Moreover, we found that the AD values in the tumor zone as well as the Cs and Cl values in WM adjacent to edema allowed for the differential diagnosis of low- and high-grade gliomas. Through ROC curve analysis, the best DTI metric was Cs in WM adjacent to edema in differentiating low-grade from high-grade gliomas. The aforementioned DTI metrics may be supplementary for the traditional use of ADC, FA, and MD markers to distinguish between low- and high-grade gliomas.

To date, most studies have evaluated the diffusion properties in low- and high-grade gliomas using ADC, FA, and MD. Piyapittayanan et al. (2013) reported that the ADC values, minimal ADC values, and ADC ratios of solid tumor regions might be critical for distinguishing high-grade from low-grade gliomas. Additionally, Inoue et al. (2005) found higher FA and lower MD values in high-grade gliomas compared with those in low-grade gliomas, and confirmed that FA and MD values might be useful for differentiating high-grade from low-grade gliomas. Our current study mainly focused on determining the AD, RD, Cp, Cs, and Cl values in glioma grading to compensate for those traditional DTI metrics. In this study, there were significant differences in the AD values between the tumor zone and NAWM in high-grade groups, while no significant differences were observed in the low-grade groups. AD represents the microscopic water movement parallel to the axonal tracts and is linked with axonal damage (Budde et al., 2009). AD in low-grade glioma tumor zones was significantly higher than that in high-grade gliomas but no significant difference was detected for RD, which was in accordance with a study by Chen F. et al. (2012). However, other studies demonstrated that both AD and RD values were significantly higher in low-grade gliomas compared to those in high-grade gliomas (Yuan et al., 2008; Server et al., 2014).

We also observed that these five metrics in the peritumoral edema zone revealed no significant differences between low- and high-grade gliomas. Kaczmarek et al. (1999) demonstrated that invasion is not only simply due to expansion by growth or a passive distribution of tumor cells but also the active translocation of tumors cells through cellular and extracellular matrix barriers of normal host tissue. Furthermore, Bordignon et al. (2006) reported great variability in the biological behavior of gliomas according to the neuroaxis spreading patterns. Low-grade glioma neuroaxis dissemination rates range from 3.7 to 5.3% (Figueiredo et al., 2003) while high-grade rates range from 10 to 27% (Misaki et al., 2001).

The DTI shape parameters (Cp and Cs) and the linear parameter (Cl) were also measured. Cp indicates the planar tensor regions, Cs suggests the isotropic tensor, and Cl specifically highlights the tubular tensor regions (Wang et al., 2011). Wang et al. (2011) reported that the Cl and Cp values of brain metastases are significantly lower than those of glioblastomas, and they are likely to differentiate glioblastomas from metastases as non-invasive measurements). Moreover, Kumar et al. (2008) reported that Cp and Cl may have advantages over FA for differentiating true from pseudo WM tracts inside an abscess cavity. In the present study, the Cp values in the tumor and peritumoral edema zones of low-grade gliomas were slightly lower than those from high-grade gliomas. The Cs and Cl values in WM adjacent to edema were significantly different between the low- and high-grade gliomas. Ma and Song (2013) reported that the area under the curve of the combined three parameters (Cs, Cl, and Cp) from the immediate peritumoral region was 0.81, which included 80% specificity and 86% sensitivity, which might make them become the best evaluated parameters for brain tumors (Cortez-Conradis et al., 2013). Importantly, anisotropy changes are associated with the macroscopic organization of tumor cells (Kim et al., 2008; Lope-Piedrafita et al., 2008). When implanting human glioma cells into a rodent brain, most glioma cells were associated with blood vessels and move to invade the unaffected brain. This leads to a breakdown of the blood brain barrier (BBB) and disrupts the neurovascular unit (Zagzag et al., 2000; Farin et al., 2006).

An effective method to evaluate the performance of diagnostic tests is ROC curve analysis, which is defined as a plot of test sensitivity (or true positive fractions as the *y* coordinate) versus 1-specificity (or false positive fractions as the *x* coordinate) (Metz, 1986; Zweig and Campbell, 1993; Hajian-Tilaki, 2013). In this study, the image diagnostic evaluation was done using a parametric ROC analysis. ROC results can give a series of right and wrong acceptance rates, while traditional diagnosis methods provide only a correct recognition rate (Hajian-Tilaki, 2013). The correct recognition rate changes if the false acceptance rate changes; therefore, traditional method cannot accurately describe the image diagnosis effect. In the ROC curve analysis utilized in this study, the best of these DTI metrics that enabled distinction between low- and high-grade gliomas was Cs in the WMZ close to edema, followed by Cl in WMZ close to edema. AD in the tumor zone had the worst performance among these three DTI metrics.

It should be noted that this study has several limitations. First, this is a retrospective case control study with a limited sample size. A larger sample size is required to confirm the relationships between these DTI metrics and glioma grading in further studies. Moreover, it is difficult to accurately determine the tumor margin, edema and the WM adjacent to edema. The histologic specimens did not necessarily come from the sites where the DTI metrics were measured.

## Conclusion

This study demonstrated that AD in the tumor zone and Cs and Cl in WM adjacent to edema provide additional information to better classify gliomas. These metrics may be complementary to the traditional tensor metrics, such as ADC, FA, and MD. A comprehensive evaluation of these DTI metrics can be used as non-invasive reliable biomarkers in glioma grading.

## Author Contributions

LJ and C-YX designed the experiments, collected the data, performed the analysis, and wrote the paper. QX, JS, and HC helped collect the data and perform the analysis. Y-CC and XY contributed to the design of the experiment and the manuscript revision.

## Conflict of Interest Statement

The authors declare that the research was conducted in the absence of any commercial or financial relationships that could be construed as a potential conflict of interest.
